# Comparison of in-person vs. remote administration of cognitive screening tools for people with ALS

**DOI:** 10.1007/s10072-024-07661-y

**Published:** 2024-07-01

**Authors:** Lyndsay Didcote, Silia Vitoratou, Ammar Al-Chalabi, Laura H. Goldstein

**Affiliations:** 1 Department of Psychology, Institute of Psychiatry, Psychology and Neuroscience, King’s College London, London, UK; 2 Department of Biostatistics and Health Informatics, Institute of Psychiatry, Psychology and Neuroscience, King’s College London, London, UK; 3 Department of Basic and Clinical Neuroscience, Maurice Wohl Clinical Neuroscience Institute, King’s College London, London, UK; 4 Department of Neurology, King’s College Hospital NHS Foundation Trust, London, UK

**Keywords:** Amyotrophic Lateral Sclerosis, Cognition, Screening tools, Psychometrics, Telehealth

## Abstract

**Objective:**

This study investigated whether cognitive screening tools used for people with amyotrophic lateral sclerosis (pwALS) are affected by the screen being administered face-to-face or remotely online. It also investigated whether demographic variables predicted total cognitive screen scores.

**Methods:**

The cognitive component of the Edinburgh Cognitive and Behavioural ALS Screen (ECASc), the cognitive component of the ALS Cognitive Behavioural Screen (ALS-CBSc), and the Mini Addenbrooke’s Cognitive Examination (Mini-ACE) were administered to 41 pwALS and 41 controls face-to-face. Versions of the cognitive screens designed to be administered remotely were administered to 57 pwALS and 44 controls via videoconferencing methods.

Backwards stepwise linear regressions were conducted to assess whether total scores on the ECASc, ALS-CBSc, and Mini-ACE scores were predicted by administration mode (face-to-face or remote) or demographic variables.

**Results:**

Mode of administration significantly affected scores on the ECASc and ALS-CBSc; remote administration was associated with better total scores. Administration mode did not significantly affect Mini-ACE scores. All cognitive screens were significantly affected by IQ scores; higher IQ scores predicted better screening tool scores. Only ECASc scores were significantly affected by age, with older age predicting poorer scores. Being female was associated with better Mini-ACE scores; sex did not predict ECASc and ALS-CBSc scores.

**Conclusions:**

Our results suggest that videoconferencing versions of the ECASc and ALS-CBSc may function differently to the original, face-to-face versions. There are advantages to using remote versions of cognitive screening tools but clinicians and researchers who use them should consider that they may not yield equivalent scores.

**Supplementary Information:**

The online version contains supplementary material available at 10.1007/s10072-024-07661-y.

## Introduction

Amyotrophic Lateral Sclerosis (ALS) is a life-limiting condition, characterised by progressive neurodegeneration. It can cause limb and bulbar weakness, resulting in difficulties with mobility, speaking, and swallowing [1, 2]. Approximately 50% of people with amyotrophic lateral sclerosis (pwALS) exhibit cognitive or behavioural changes, and of these about 15% present with ALS-frontotemporal dementia (ALS-FTD) [3, 4]. Screening tools have been devised to detect cognitive changes in pwALS, such as the cognitive component of the Edinburgh Cognitive and Behavioural ALS Screen (ECASc) [5] and the cognitive component of the ALS Cognitive Behavioural Screen (ALS-CBSc) [6]. It has also been proposed that the Mini-Addenbrooke’s Cognitive Examination (Mini-ACE) [7] (Table 1), which was originally devised for detecting cognitive impairment in people with dementia, could be applied to pwALS. These cognitive screening tools differ in the tasks involved, cognitive domains assessed, and the extent of evidence for their reliability and validity [8]. They also differ in the extent to which they accommodate the motor and speech impairments [1, 2] that may be present in pwALS.

**Table 1:** Descriptions of cognitive screening tools as developed for face-to-face administration

Screening tool	Description	Time to administer (minutes)	Scores^a^	Cut-off scores^b^
ECASc [5]	Assesses cognitive function that is both ALS-specific (executive function (including social cognition), verbal fluency, and language) and non-ALS-specific (memory and visuospatial function). All cognitive tasks can be completed by a person with ALS if they can give their answers orally or in writing. In timed fluency tasks, participants are given two minutes to complete them if they give their answers in writing but only one minute if they answer orally	15	Min: 0 Max: 136	ALSci: ≤ 105/136
ALS-CBSc [6]	Cognitive domains assessed by this measure include verbal fluency; concentration; attention; mental tracking; and monitoring. Instructions for administering the command task for the attention assessment say that if a patient is unable to follow instructions with their finger, the movement can be substituted with eye movement. However, it is not possible to substitute “touch your shoulder” and “make a fist” with eye movements without changing the task. Most tasks ask for oral responses, though it is possible to complete most tasks in writing (concentration task; mental addition/language task which assesses attention; the months task which assesses tracking/monitoring) but it is unclear whether this changes the nature of the task. For the initiation and retrieval task, the time limit is the same irrespective of whether answers are given orally or in writing. Therefore, it does not account for the extra time it takes to write rather than say a word (while the ECASc contains the same task and doubles the time limit for those who write their answers)	5	Min: 0 Max: 20	ALSci: < 17/20 ALS-FTD: ≤ 10/20
Mini-ACE [7]	A condensed version of the Addenbrooke’s Cognitive Examination – III [36], developed to comply with the short assessment time available during clinics. It is not specific to ALS and assesses attention, memory, fluency, visuospatial function, and memory Some tasks are intended to be spoken but can be written, such as the memory and fluency task, though it is not clear if this changes the nature of the task. The fluency task is timed and there is no time adjustment for those who write their responses. The visuospatial function task cannot be completed if the person with ALS cannot write	5	Min: 0 Max: 30	ALS-FTD: ≤ 25/30^c^

^a^ For all cognitive screening tools, lower scores indicate poorer cognitive functioning. ^b^ Cut-off scores apply to total scores on the cognitive screening tools. ^c^ The Mini-ACE cut-off score of ≤ 25/30 was generated using a dementia sample [7] and was later used with a sample of pwALS by the same author [37]

During the COVID-19 pandemic, telehealth techniques were employed to maintain contact between pwALS and clinicians, minimise face-to-face interactions to limit the spread of COVID-19, improve quality of life for caregivers, and limit anxiety and depression related to the pandemic [9]. This prompted a need to develop methods of assessing patients remotely, and this has included the assessment of cognitive change in pwALS which has typically been conducted face-to-face.

However, concerns have been raised about administering cognitive tests remotely that involve visual stimuli [10]. Without validation of remote versions of these face-to-face tests, it cannot be assumed that they function exactly as the original face-to-face versions [11], especially when stimuli are shared through desktop projectors or using screen-sharing functions within videoconferencing applications. Telephone versions of the ALS-CBS [6] have been validated but the telephone versions [12, 13] have required adaptations that involved the removal of items that require visual assessment and their replacement by tasks that could be assessed using this remote assessment modality.

A recent study, which investigated the remote version of the ECASc delivered through videoconferencing methods, revealed that among participants without ALS, no significant differences in total scores were observed between the remote and original in-person versions of the ECASc. Good agreement was found between the two assessment methods [14].

Remote, videoconferencing versions of the ECASc, ALS-CBSc and Mini-ACE have yet to be tested in pwALS within a single study. This study aims to assess i) whether administering the cognitive screening tools remotely, rather than in-person, affects total cognitive screen scores, and ii) whether demographic variables (age, sex, IQ) predict total cognitive screen scores.

## Methods

Ethical approval was obtained from the London-Dulwich Research Ethics Committee and the Health Research Authority (Reference 18/LO/1257). A remote version of the ECASc [14] had already been developed at the time and permission was obtained to use that version.

This research was carried out before and during the coronavirus pandemic, resulting in two samples, one where research was conducted in person, face-to-face (F2F) between 29 January 2019 and 10 March 2020 and one non-overlapping sample where assessments were conducted remotely, online between 11 September 2020 and 30 June 2021.

### Participants

Participants with ALS were recruited from four National Health Service (NHS) Motor Neuron Disease (MND) clinics. Healthy control participants were local to the Institute of Psychiatry, Psychology and Neuroscience (King’s College London) in Camberwell, London and responded to physical advertisements posted in public spaces and in online local forums.

Recruitment of pwALS and healthy controls in the remote condition was conducted online as well as from the above MND clinics. Participants responded to recruitment advertisements placed on the MND Association website, MND Association newsletters, classified advertisement websites, and social media.

Participants were included if they had a diagnosis of El Escorial defined ALS (but excluding progressive muscular atrophy and primary lateral sclerosis); were at least 18 years old and were able to give informed consent. In the remote condition, participants were also required to have access to a tablet (minimum diagonal screen length of 10 inches), laptop, or desktop PC with speakers, microphone, webcam, and a wi-fi connection that could facilitate videoconferencing.

ALS participants were excluded if they used non-invasive ventilation (NIV) during the day. All participants were excluded if they were aged over 75; had a history of significant head injury, stroke, or neurodegenerative disease (other than ALS with or without FTD for the ALS group); were receiving treatment for any other life-limiting illness; or if English was not their native language. They were also excluded if they scored > 10 on the Epworth Sleepiness Scale (ESS; [15]), indicating an excessive level of daytime sleepiness.

### Process of administering measures to participants

In the face-to-face condition, all participants with ALS were visited at home for a period of two-to-four hours, often with breaks within testing sessions to minimise fatigue. Stimuli were presented to participants on paper. In the remote condition, visual stimuli (for which there was no permission to use scanned material) were presented to participants via videoconferencing methods (via Skype or Zoom) using a document camera (visualiser; Thustand USB Document Camera 8MP/2448P) that functioned as an overhead projector and was displayed as a secondary webcam.

Background and demographic information were collected first. This included the assessment of functional status using the Revised ALS Functional Rating Scale (ALSFRS-R; [16]). Following this, the three cognitive screening tests (Table 1) were administered to participants in a pseudorandomised order with short breaks in-between the screening tests. A pseudorandomised order of presentation was adopted in both the face-to-face and remote conditions.

An estimate of IQ was made using the Test of Premorbid Functioning [17] during testing sessions.

### Development of remotely administered measures

The existing remote version of the ECASc [14] was obtained from the developers and the other measures were converted into online versions, with permission from authors.

Some changes to the response format of the cognitive screens were made in the remote condition so that participants could communicate their answers using a tablet/laptop/desktop PC. Where participants could select their choice of stimuli in the face-to-face condition by pointing, they instead named/described their selection or described the position of the selected item from among the other stimuli items in the remote condition. For written verbal fluency tasks in the remote condition, participants wrote their answers on paper as they would have done in the face-to-face condition and then held their paper up to the webcam so that the number of words that they had generated could be summed for the generation of a verbal fluency index [18]. For tasks of alternating numbers and letters in the ECASc and ALS-CBSc, when the participants gave their answers in writing, one set (i.e., a number and a letter) was written on a sheet of paper which was then turned over and the next set was written on another sheet of paper and so on. Answers given in writing in the face-to-face condition that were not for specific writing tasks (i.e., tasks that are usually spoken but can be written if bulbar symptoms are present) were typed in the chat function of the videoconferencing software in the remote condition.

### Incomplete data

Where it was not possible for ALS participants to give responses to tasks within the ALS-CBSc (due to muscle wasting preventing the raising of arms or making a fist) and Mini-ACE (due to muscle wasting preventing the ability to draw a clock), the full score for the task was awarded. In a wider research project by the current authors to be described elsewhere, total screen scores were used to classify participants as being cognitively impaired. Simply awarding no points for a task in this scenario was not appropriate as this might result in participants falling below the total score cut-off, classifying them as cognitively impaired without objective evidence.

Similarly, prorating scores was not deemed sensible. The screens appear to assess several cognitive domains and may include only one task per domain. Unidimensionality of the screens has not been demonstrated using factor analysis.

We chose not to exclude from our data analyses participants who were unable to complete all task items since this was not prespecified in our eligibility criteria. It would also have affected statistical power and limited the generalisability of our sample of ALS participants to the wider population of pwALS who might be required to undergo screening cognitive assessments. Sensitivity analyses were conducted to assess whether incomplete data on the ALS-CBSc and Mini-ACE affected results.

### Statistical analysis

The sample used in this research is part of a wider sample collected for a broader research project. A posteriori power analysis confirmed appropriate power for the regressions carried out in this study.

Backwards stepwise linear regression models were used to assess whether mode of administration (face-to-face or remote administration) predicted total ECASc, ALS-CBSc and Mini-ACE screen scores.

To identify differences in demographic variables and screening test scores between ALS and healthy control samples and differences between face-to-face and remote samples, *t*-tests, Mann–Whitney-*U* tests, Chi-square tests, and Fisher’s Exact tests were used. Where there were significant differences between samples, these variables were entered as covariates in the linear regression models.

In the regression models, variables were chosen to be removed individually at each step based on p values (p > 0.05), with variables that had larger non-significant p values being removed first and the model recalculated before any other variables were removed. The variable ‘group’ (ALS or control) was not removed, regardless of whether or not it was a significant predictor of total screening tool score, in order to control for this in all analyses.

For bivariate group comparisons (i.e., *t*-tests, Mann–Whitney *U* tests, chi-square tests), casewise deletion was employed. For multiple linear regressions, cases that had missing data for any variable were excluded (listwise deletion).

Analysis was carried out using SPSS v27; p values < 0.05 were considered significant.

## Results

### Sample size

The total number of pwALS that participated in the study was 98 (see Fig. 1–4 in Online Resource 1). Of these, 41 were tested face-to-face and 57 were tested remotely (see Table 2). The total number of control participants tested was 85, with 41 tested face-to-face and 44 tested remotely (see Table 2).

**Table 2:** Available samples for each cognitive screening tool

		F2F^a^ (n)	REM^b^ (n)	Combined (n)
ECASc				
	Control	41	44	85
	ALS^c^	39	57	96
ALS-CBSc				
	Control	40	44	84
	ALS	38	57	95
Mini-ACE				
	Control	41	44	85
	ALS	40	57	97

^a^ F2F = face-to-face, ^b^ REM = remote. ^c^ ALS = Amyotrophic lateral Sclerosis. One ALS participant, tested face-to-face, did not complete the ECASc because the researcher ended data collection early due to lack of engagement of the participant. A second did not complete the ALS-CBSc because the face-to-face testing environment did not feel safe to the researcher and so data collection was ended early. A face-to-face ALS participant and a face-to-face control chose not to complete the ALS-CBSc, although they completed all other measures. One ALS participant in the face-to-face condition gave consent and decided not to complete any of the tests (no data was available for the ECASc, ALS-CBSc, and Mini-ACE)

### Characteristics and clinical variables

ALS participants in the face-to-face and remote testing conditions were broadly comparable (Table 1 in Online Resource 1). No significant differences were found in the ratios of females to males, age, years of education, ALSFRS-R scores [16], NIV use, method of communication, disease duration, time from symptom onset to diagnosis, and limb-onset vs bulbar-onset between face-to-face and remotely tested ALS samples (Table 1 in Online Resource 1). There was, however, a significant difference in IQ estimates between remotely and face-to-face tested ALS participants (p = 0.002) with face-to-face tested participants having higher mean IQ estimates (although both groups’ estimated IQ still fell within the Average range of IQ classifications; Table 1 in Online Resource 1).

As a result of the coronavirus pandemic and associated lockdowns, face-to-face data collection was stopped abruptly, resulting in control and ALS samples that did not have comparable demographics. Within the face-to-face condition, years of education and IQ estimates were similar. However, there was a significant difference in age (*p* < 0.001; higher in ALS group) and in the ratio of females to males (p = 0.038; higher female proportion among controls) (Table 2 in Online Resource 1).

For the remotely tested samples, age, years of education and sex were comparable (Table 2 in Online Resource 1). ALS participants had higher average IQ estimates than control participants (*p* < 0.001). Due to the between groups difference in IQ sores, but not in years of education, IQ was investigated as a predictor of screening test scores in the current study.

Where ALS and control sample demographics were not comparable, these differences were assessed further in regression analyses.

### Regression models

Multiple regression analyses were used to assess whether administration mode (face-to-face or remote administration) and demographic variables significantly predicted total scores on the cognitive screening tools.

#### Mode of administration

Simple differences in cognitive screen scores between the face to face and remote samples are shown in Table 3 in Online Resource 1.

Our regression analyses showed that for the ECASc model, sex did not significantly predict ECASc total scores and so was removed. The final model explained a statistically significant amount of the variance in ECASc scores and all of the included predictor variables were individually significant (Table 3). Administration mode was a significant predictor of ECASc scores; higher scores were associated with remote administration.

**Table 3:** Statistics for individual variables entered into multiple regression models to assess the ability of participant group, administration mode, and demographic factors to predict cognitive screening tool scores

Outcome: ECASc score F(4, 152) = 11.947, *p* < 0.001, R^2^ = 0.239		
*Predictors*	***B (SE)***	***t***
(constant)	72.4 (11.4)	***6.380
Administration mode^a^	6.2 (1.5)	***4.183
Group^b^	-3.4 (1.5)	*-2.270
IQ^c^	0.5 (0.1)	***4.737
Age	-0.2 (0.1)	*-2.099
Sex	Omitted^d^	Omitted
Outcome: ALS-CBSc score F (3, 151) = 10.803, *p* < 0.001, R^2^ = 0.177		
*Predictors*	***B (SE)***	***t***
(constant)	8.3 (2.8)	**2.954
Administration mode	1.3 (0.4)	***3.549
Group	-1.1 (0.4)	**-2.974
IQ	0.1 (0.03)	**3.213
Age	Omitted	Omitted
Sex	Omitted	Omitted
Outcome: Mini-ACE score F(4, 153) = 5.965, *p* < 0.001, R^2^ = 0.135		
*Predictors*	***B (SE)***	***t***
(constant)	13.8 (3.4)	***4.010
Administration mode	0.9 (0.5)	1.862
Group	-0.1 (0.5)	-0.197
IQ	0.1 (0.03)	***3.999
Age	Omitted	Omitted
Sex	1.2 (0.5)	**2.648

^***^p < 0.001, **p < 0.01, *p < 0.05. ^a^ Administration mode categories: tested face-to-face/tested remotely. ^b^ Group categories: ALS/control. ^c^ IQ estimated using Test of Premorbid Functioning: full-scale IQ, scored 42–127. Estimated IQ was selected instead of years of education as there were significant differences in estimated IQ, but not years of education, between different samples (between ALS and control groups in remote and combined samples and between face-to-face and remote ALS samples). It was decided that it was more important to control for estimated IQ, rather than years of education, by including it in the regression models. ^d^ Omitted variables were tested but omitted from the final model, following a backwards stepwise procedure

In the ALS-CBSc model, sex and age were not significant predictors of ALS-CBSc total scores. Sex was removed from the model first and the model was re-calculated. Following this, age was removed. The final model explained a statistically significant amount of the variance in ALS-CBSc scores. Administration mode was a significant predictor of ALS-CBSc scores; higher scores were associated with remote administration. All independent variables included in the final model significantly predicted ALS-CBSc scores (Table 3).

For the Mini-ACE model, age was not a significant predictor of Mini-ACE scores and so was removed. Administration mode was not a significant predictor of Mini-ACE scores.

The Attention – Commands task in the ALS-CBSc was not completed by 8/38 (21.1%) ALS participants in the face-to-face condition and by 9/57 (15.8%) in the remote condition. The Clock Drawing task in the Mini-ACE was not completed by 19/40 (47.5%) ALS participants in the face-to-face condition and by 16/57 (28.1%) in the remote condition. Simple differences in cognitive screen scores between the face to face and remote samples with incomplete data removed are shown in Table 4 in Online Resource 1. Sensitivity regression analyses indicated that removal of the incomplete items from the ALS-CBSc and Mini-ACE had no substantial effect on results (see Table 5 in Online Resource 1) and again mode of administration predicted scores on the ECASc and ALS-CBCc but not on the Mini-ACE.

#### Demographic predictors of ECASc, ALS-CBSc and Mini-ACE scores

Estimated IQ scores predicted ECASc, ALS-CBSc, and Mini-ACE scores (higher IQ scores were associated with higher screening tool scores). Age predicted ECASc scores (higher age was associated with lower ECASc scores), but not ALS-CBSc or Mini-ACE scores. Sex predicted Mini-ACE scores (being female was associated with higher Mini-ACE scores) but not ECASc and ALS-CBSc scores. In our sensitivity analyses, where the incomplete ALS-CBSc and Mini-ACE items were removed, the pattern of demographic predictors was essentially unchanged.

## Discussion

This study assessed the performance of remote administrations of the ECASc, ALS-CBSc and Mini-ACE in comparison to their original face-to-face versions and assessed which demographic variables affected cognitive screening tool total scores.

Mode of administration was found to be a significant predictor of ALS-CBSc and ECASc scores, with higher scores found in the remote condition, but not of Mini-ACE scores (Table 3). This was found also to be the case in our sensitivity analyses where items that could not be completed by pwALS due to motor problems were removed from the analyses. Elsewhere, in a cohort without ALS, comparable results for face-to-face and remote administration were observed for ECASc total scores. The authors concluded that their results suggested the remote and face-to-face versions of the ECASc are interchangeable, generating equivalent scores [14]. This contrasts with the present study’s findings and may be due to the difference in statistical methods employed.

It may also be possible that mode of administration was predictive of scores on the ALS-CBSc and ECASc (although not the Mini-ACE) because of a confound of poor demographic comparability between ALS and control samples in the face-to-face condition (due to pandemic-related restrictions on data collection) despite appropriate comparability in the remote condition. Thus, individual differences may account for discrepancies in screening tool scores between the face-to-face and remote conditions; however, for this to be true overall, it would be expected that mode of administration would also have predicted Mini-ACE scores, which was not found to occur. No significant differences in screening test scores were found between face-to-face and remote conditions in the ALS sample (Tables 3 and 4 in Online Resource 1). This leaves open the possibility that further investigations are required into the comparability of face-to-face and remote versions of these tests.

A number of participants were unable to complete a task in either the ALS-CBSc and/or the Mini-ACE. For these tasks, arm strength and dexterity in the hands were required, something that is often a problem for pwALS. These physical demands of the ALS-CBSc and Mini-ACE highlighted the suitability of the ECAS for individuals with ALS where task completion can be undertaken interchangeably via speech or writing and avoids the scenario where testing is deemed impossible for patients.

Findings of the effects of age and estimated IQ on cognitive screen scores support other reported findings regarding the impact of age and years of education/IQ. Alternative age- and education-based cut-off scores were developed for German and Italian versions of the ECASc once effects of age and years of education were found [19, 20]. One study found ECASc scores were predicted by IQ and by age [21]. ALS-CBSc scores have also been shown to correlate with level of education [6]. In the literature, no correlation between the ALS-CBSc scores and age has been reported, as in the current study.

The ECASc may benefit from different cut-off scores for different age ranges [19, 21, 22]. It may also be useful to have different cut-off scores for different IQ estimate ranges and for different modes of administration (face-to-face and remote conditions) for all cognitive screens. However, it may be challenging to apply estimated IQ specific cut-off scores given that estimated IQ based on reading tests may be impacted by language-based difficulties in pwALS. Indeed, some studies have reported, albeit nonsignificant, trends for premorbid IQ estimates based on reading to be lower in pwALS than in controls [23, 24].

During the coronavirus pandemic, there was a need for clinical activities to continue remotely [9]. Remote versions of the cognitive screening tools may be useful in the future during other periods of time that require pwALS to isolate for health reasons. The remote versions of screening tools are likely to be useful for pwALS, their caregivers and clinicians in general. The remote administration of screening tools can potentially reduce fatigue in pwALS and caregivers by reducing travel, reducing time spent in clinics, and enabling both to be in a comfortable, more relaxed environment at home, provided that distractions can be minimised.

In addition, there is a consensus that complementary telehealth may be useful for pwALS, their caregivers and clinicians [25–30] and the remote screening of cognitive change may be a good candidate for this.

A potential limitation to the usefulness of remote versions is the technological literacy of some pwALS, particularly older individuals [11], who may struggle with using the software needed for testing via videoconferencing methods. The cognitive screens will also not be suitable for patients with poor internet connections, or who have computer equipment with only small screen display options. Technical issues could affect performance on the tests (e.g., a connection lag leading to a need to repeat a stimulus or prompt, a poor connection making it challenging to see the stimulus clearly). The need to repeat some prompts due to internet variability may make tasks easier leading to higher scores than without such internet issues. Development of guidance for what to do in these scenarios will be important. If the remote administration of these screening tools is widely adopted, application of in-person versions must still be available for those for whom remote assessments are not clinically suitable [31].

While remote versions of some cognitive screening tools administered via telephone have been developed and tested (for the ALS-CBSc and Mini-ACE [12, 13, 32]), the potential identified in the current study for remote versions of the cognitive screens to be administered via videoconferencing methods greatly reduces the need for items to be altered to suit the format of administration. In videoconferencing versions, the same stimuli as in the original face-to-face screens are administered, just via a PC or tablet screen, maintaining the structure of the screening tools. Results from the current study suggest that the remote versions of cognitive screens may, nonetheless, function differently to face-to-face versions.

### Strengths and limitations

This study is the first to investigate the utility of remote videoconferencing versions of the ALS-CBSc and Mini-ACE and extend evaluation of the remote version of the ECAS [14]). However, it is not without limitations.

IQ scores were not comparable between ALS and control participants in the remote sample, though the variable ‘years of education’ was comparable between the two groups. While this may reflect an effect of ALS on language-based tasks including reading [33, 34] making it possible that IQ scores may have been influenced by other aspects of ALS-related cognitive change, no significant difference was found between ALS participants and controls in the face-to-face condition and IQ scores were controlled for in analyses where possible.

An issue with introducing the assessment of the utility of remote versions of the screening tools during the study, in response to the coronavirus pandemic restrictions, without planning the remote condition before study activity began, is that it resulted in independent samples being used in the remote and face-to-face conditions for the evaluation of cognitive impairment, and raised the possibility that individual differences accounted for the findings regarding mode of administration. A repeated measures design, where the same sample participated in both the remote and face-to-face conditions in a counterbalanced order, would have controlled for these individual differences and have allowed a purer evaluation of the effect of mode of administration.

### Recommendations for future research

Future research should investigate how well the remote versions of screens compare to the original face-to-face versions of the screens using repeated measures methodology as has been undertaken in healthy individuals for the ECAS [14]. This will control for the possibility of a confounding effect of individual differences between the remote and face-to-face samples that may have occurred in the current study.

Administration instructions for cognitive measures should consider the potential that the measure may be administered digitally and offer guidance as to what should happen in cases of poor internet connection where questions/prompts need to be repeated, or where distractions may occur in the home setting.

Authors of cognitive measures to be used remotely should also consider providing guidance for participants/patients who are limited by technological literacy. This may enable more patients in remote clinical settings to complete cognitive measures, which may reduce fatigue, time, and costs related to travel [26] and be more convenient for patients [35]. Offering guidance to research participants about remote cognitive testing may also increase recruitment/engagement rates as this guidance would reduce the limitation of poor technological literacy and remove travel and its associated fatigue and inconvenience for pwALS.

## Supplementary Information

Below is the link to the electronic supplementary material.

Supplementary file1 (DOC 133 KB)

## Data Availability

Anonymised data may be available on reasonable request.
