# Supplementary material for: Comparison of in-person vs. remote administration of cognitive screening tools for people with ALS
Source: Neurol Sci. 2024 Jul 1;45(11):5309–17. doi: 10.1007/s10072-024-07661-y (PMC11470852; doi:10.1007/s10072-024-07661-y)
Supplement: Supplementary file 1 — Supplementary file1 (DOC 133 KB) [file 10072_2024_7661_MOESM1_ESM.docx]

Comparison of in-person vs remote administration of cognitive screening tools for people with ALS: Online Resource 1

Caption describing content of this file: Additional tables describing differences in demographic variables, screening tool scores, incomplete ALS participant data and figures showing recruitment pathways.

Table 1 – Differences in demographic variables in the ALS sample between face-to-face and remote testing conditions

|  | | **n (%)** | | ***χ^2^ (df)*** |
| --- | --- | --- | --- | --- |
|  |  | **F2F^a^** | **REM^b^** |  |
| Sex | Male | 19 (46.3) | 37 (64.9) | 3.358 (1) |
|  | Female | 22 (53.7) | 20 (35.1) |  |
| NIV^c^ | Yes | 6 (15.0) | 7 (12.3) | 0.150 (1) |
|  | No | 34 (85.0) | 50 (87.7) |  |
| Onset | Limb | 33 (82.5) | 45 (79.0) | 0.188 (1) |
|  | Bulbar | 7 (17.5) | 12 (21.1) |  |
| Method of communication | Spoken only | 21 (52.5) | 25 (43.9) | 1.723 (2) |
|  | Written only | 2 (5.0) | 7 (12.3) |  |
|  | As requested by tests | 17 (42.5) | 25 (43.9) |  |
|  | | **Median (IQR)** | | ***U (z)*** |
|  |  | **F2F n = 40** | **REM n = 57** |  |
| Disease duration (months) | | 27.0 (45.0) | 33.0 (15.5) | 1082 (-0.6) |
| Time between onset and diagnosis (months) | | 13.0 (16.5) | 9.0 (18.0) | 1014.5 (-1.1) |
|  | | **Mean (SD)** | | ***t (df)*** |
|  |  | **F2F n = 40** | **REM n = 57** |  |
| Age | | 64.3 (8.6) | 60.6 (9.7) | 2.0 (95) |
| Years of education | | 15.7 (4.3) | 16.3 (3.0) | -0.7 (65) |
| IQ estimate^d^ (possible range 42-127) | | 106.2 (8.2) | 100.8 (6.6) | **3.2 (76)^e^ |
| ESS score^f^ (possible range 0-24) | | 3.8 (2.1) | 4.3 (2.8) | -0.7 (77) |
| ALSFRS-R^g^ (possible range 0-48) | | 34.9 (7.1) | 34.1 (8.1) | 0.5 (94) |

**p < 0.01. ^a^ F2F = face-to-face, ^b^ REM = remote. ^c^ NIV = non-invasive ventilation. ^d^ IQ estimated using Test of Premorbid Functioning: full-scale IQ. ^e^ Cohen’s d was above 0.7 for the significant t-test. ^f^ ESS = Epworth Sleepiness Scale [1]: participants who scored >10 were excluded. ^g^ ALSFRS-R = Amyotrophic Lateral Sclerosis Functional Rating Scale – Revised [2].

Table 2 – Differences in demographic variables between ALS and control participants.

|  | | | **Mean (SD)** | | ***t (df)*** |
| --- | --- | --- | --- | --- | --- |
|  |  |  | **ALS^a^**  F2F^b^ n = 40  REM^c^ n = 57 | **Control**  F2F n = 41  REM n = 44 |  |
| Age | Face-to-face | | 64.3 (8.6) | 56.2 (10.3) | ***-3.9 (79) |
|  | Remote | | 60.6 (9.7) | 63.0 (7.7) | 1.4 (99) |
|  | Combined | | 62.1 (9.4) | 60.0 (9.6) | -1.7 (180) |
| Years of education | Face-to-face | | 15.7 (4.3) | 17.1 (2.9) | 1.7 (68) |
|  | Remote | | 16.3 (3.0) | 16.8 (3.0) | 0.9 (99) |
|  | Combined | | 16.0 (3.6) | 17.0 (2.9) | 1.9 (179) |
| IQ score^d^ (possible range 42-127) | Face-to-face | | 106.2 (8.2) | 108.8 (7.9) | 1.4 (75) |
|  | Remote | | 100.8 (6.6) | 106.1 (5.7) | ***3.8 (79) |
|  | Combined | | 103.3 (7.8) | 107.5 (7.0) | **3.4^e^ (156) |
|  |  | | **N(%)** | | ***χ^2^ (df)*** |
|  |  |  | **ALS**  F2F n = 40  REM n = 57 | **Control**  F2F n = 41  REM n = 44 |  |
| Sex | Face-to-face | |  |  |  |
|  |  | Male | 19 (46.3) | 10 (24.4) | *4.321 (1) |
|  |  | Female | 22 (53.7) | 31 (75.6) |  |
|  | Remote | |  |  |  |
|  |  | Male | 37 (64.9) | 29 (65.9) | 0.011 (1) |
|  |  | Female | 20 (35.1) | 15 (34.1) |  |
|  | Combined | |  |  |  |
|  |  | Male | 56 (57.1) | 39 (45.9) | 2.312 (1) |
|  |  | Female | 42 (42.9) | 46 (54.1) |  |

***p < 0.001, **p < 0.01, *p < 0.05. a ALS = Amyotrophic lateral Sclerosis. b F2F = face to face, c REM = remote. d IQ assessed using Test of Premorbid Functioning: full-scale IQ. Unless e Cohen’s d is moderate (between 0.5 and 0.6). Unless otherwise stated, Cohen’s d was above 0.8 for significant t-tests.

Table 3 - Differences in cognitive screen scores between face to face and remote samples.

|  | | Median (IQR) n | | *U(z)* |
| --- | --- | --- | --- | --- |
|  |  | F2F^a^ | REM^b^ |  |
| ALS sample | |  |  |  |
|  | ECASc | 113 (12.1) n=39 | 116 (9.8) n=57 | 940.5(-0.28) |
|  | ALS-CBSc | 17 (3.2) n=38 | 17 (2.4) n=57 | 1005.0(-0.60) |
|  | Mini-ACE | 28 (2.4) n=40 | 28 (2.2) n=57 | 1108.0(-0.24) |
| Control sample | |  |  |  |
|  | ECASc | 118 (10.5) n=41 | 122 (8.0) n=44 | 620.0(-2.48)* |
|  | ALS-CBSc | 17 (2.0) n=40 | 19 (1.5) n=44 | 564.5(-2.87)** |
|  | Mini-ACE | 29 (2.3) n=41 | 29 (4.1) n=44 | 813.5(-0.81) |

*= *p* < .05. **= *p* < .01. ^a^ F2F = face-to-face, ^b^ REM = remote.

Table 4 - Differences in cognitive screen scores between face to face and remote samples with ALS-CBSc and Mini-ACE incomplete items removed.

|  | | Median (IQR) n | | *U(z)* |
| --- | --- | --- | --- | --- |
|  |  | F2F^a^ | REM^b^ |  |
| ALS sample | |  |  |  |
|  | ECASc | 113 (12.1) n=39 | 116 (9.8) n=57 | 940.5(-0.28) |
|  | ALS-CBSc | 16 (2.3) n=38 | 16 (3.5) n=57 | 995.0(-0.68) |
|  | Mini-ACE | 23 (2.8) n=40 | 23 (2.5) n=57 | 1082.0(-0.43) |
| Control sample | |  |  |  |
|  | ECASc | 118 (10.5) n=41 | 122 (8.0) n=44 | 620.0(-2.48)* |
|  | ALS-CBSc | 16 (3.0) n=40 | 18 (3) n=44 | 564.5(-2.87)** |
|  | Mini-ACE | 24 (2.0) n=41 | 24 (1.8) n=44 | 833.5(-0.64) |

*= *p* < .05. **= *p* < .01. ^a^ F2F = face-to-face, ^b^ REM = remote.

Table 5: Sensitivity analysis: statistics for individual variables entered into multiple regression models to assess the ability of participant group, administration mode, and demographic factors to predict cognitive screening tool scores omitting items where data was not complete on the ALS-CBSc and the Mini-ACE.

| Outcome: ECASc score F(4, 152) = 11.947, p < 0.001, R^2^ = 0.239 | | |
| --- | --- | --- |
| ***Predictors*** | ***B (SE)*** | ***t*** |
| (constant) | 72.4 (11.4) | ***6.380 |
| Administration mode^a^ | 6.2 (1.5) | ***4.183 |
| Group^b^ | -3.4 (1.5) | *-2.270 |
| IQ^c^ | 0.5 (0.1) | ***4.737 |
| Age | -0.2 (0.1) | *-2.099 |
| Sex | Omitted^d^ | Omitted |
| Outcome: ALS-CBSc score^e^  F (3, 151) = 10.219, p < 0.001, R^2^ =0.169 | | |
| ***Predictors*** | ***B (SE)*** | ***t*** |
| (constant) | 7.7 (2.8) | **2.746 |
| Administration mode | 1.3 (0.4) | ***3.621 |
| Group | -1.0 (0.4) | **-2.788 |
| IQ | 0.1 (0.03) | **3.107 |
| Age | Omitted | Omitted |
| Sex | Omitted | Omitted |
| Outcome: Mini-ACE score^f^ F(4, 153) = 5.038, p < 0.001], R^2^ =0.116 | | |
| ***Predictors*** | ***B (SE)*** | ***t*** |
| (constant) | 11.4 (3.3) | ***3.469 |
| Administration mode | 0.7 (0.5) | 1.587 |
| Group | -0.2 (0.4) | -0.427 |
| IQ | 0.1 (0.03) | ***3.539 |
| Age | Omitted | Omitted |
| Sex | 1.1 (0.4) | *2.480 |

***p < 0.001, **p < 0.01, *p < 0.05. a Administration mode categories: tested face-to-face/tested remotely. b Group categories: ALS/control. c IQ estimated using Test of Premorbid Functioning: full-scale IQ, scored 42-127. Estimated IQ was selected instead of years of education as there were significant differences in estimated IQ, but not years of education, between different samples (between ALS and control groups in remote and combined samples and between face-to-face and remote ALS samples). It was decided that it was more important to control for estimated IQ, rather than years of education, by including it in the regression models. d Omitted variables were tested but omitted from the final model, following a backwards stepwise procedure. e Total ALS-CBSc score minus the Attention-Commands sub-score. f Total Min-ACE score minus the drawing task sub-score.

Figures were created in Microsoft Word version 2312.

**Fig1**: Flowchart showing recruitment pathway for the ALS participants in the face-to-face condition.

A Flowchart describing the recruitment pathway for the ALS participants in the face-to-face condition. The numbers of participants not included in the final sample due to loss of contact, and meeting exclusion criteria are given. Each of these pieces of information is given in a box with arrows connecting the boxes to the next stage of recruitment. The stages of recruitment are identified or volunteered during recruitment phase, screened against exclusion criteria, consented, and included in analysis

Included in analysis (n = 41)

Consented (n = 41)

Excluded (n = 4)

- Scored above 10 on Epworth Sleepiness scale (n = 4)

Screened against exclusion criteria (n = 45)

Contact lost prior to giving consent (n = 24)

Identified or volunteered during recruitment phase (n = 69)

Face-to-face recruited ALS^a^ sample

^a^ Amyotrophic lateral Sclerosis.

**Fig2**: Flowchart showing recruitment pathway for the control participants in the face-to-face condition.

A Flowchart describing the recruitment pathway for the control participants in the face-to-face condition. The numbers of participants not included in the final sample due to loss of contact, deciding not to participate, meeting exclusion criteria, and being poorly demographically matched with the ALS sample are given. The stages of recruitment are identified or volunteered during recruitment phase, screened against exclusion criteria, consented, and included in analysis

Identified or volunteered during recruitment phase (n = 70)

Face-to-face recruited control sample

Contact lost prior to giving consent (n = 8)

Chose not to participate prior to giving consent (n = 1)

Screened against exclusion criteria (n = 61)

Excluded (n = 2)

- Scored above 10 on Epworth Sleepiness scale (n = 1)
- Previous head injury (n = 1)

Not invited to give consent as demographic information, specifically age and sex, was not well matched with ALS^a^ sample (n = 18)

Consented (n = 41)

Included in analysis (n = 41)

^a^ Amyotrophic lateral Sclerosis.

**Fig3**: Flowchart showing recruitment pathway for the ALS participants in the remote condition.

A Flowchart describing the recruitment pathway for the ALS participants in the remote condition. The numbers of participants not included in the final sample due to loss of contact, deciding not to participate, and meeting exclusion criteria are given. The stages of recruitment are identified or volunteered during recruitment phase, screened against exclusion criteria, consented, and included in analysis

Identified or volunteered during recruitment phase (n = 109)

Remotely recruited ALS^a^ sample

Contact lost prior to giving consent (n = 20)

Chose not to participate prior to giving consent (n = 2)

Screened against exclusion criteria (n = 87)

Excluded (n = 27)

- Not based in UK (n = 2)
- Over age 75 (n = 6)
- Scored above 10 on Epworth Sleepiness scale (n = 3)
- Diagnosis of PLS^b^ (n = 10)
- Anarthric and unable to write or type (n = 6)

Consented (n = 60)

Contact lost after consent was given (n = 3)

Included in analysis (n = 57)

^a^ Amyotrophic lateral Sclerosis, ^b^ Primary Lateral Sclerosis.

**Fig4**: Flowchart showing recruitment pathway for the control participants in the remote condition.

A Flowchart describing the recruitment pathway for the control participants in the remote condition. The numbers of participants not included in the final sample due to loss of contact, deciding not to participate, meeting exclusion criteria, and being poorly demographically matched with the ALS sample are given. The stages of recruitment are identified or volunteered during recruitment phase, screened against exclusion criteria, consented, and included in analysis

Remotely recruited control sample

Identified or volunteered during recruitment phase (n = 188)

Contact lost prior to giving consent (n = 5)

Chose not to participate prior to giving consent (n = 2)

Screened against exclusion criteria (n = 181)

Excluded (n = 3)

- Scored above 10 on Epworth Sleepiness scale (n = 3)

Not invited to give consent as demographic information, specifically age and sex, was not well matched with ALS^a^ sample (n = 132)

Consented (n = 46)

Contact lost after consent was given (n = 2)

Included in analysis (n = 44)

^a^ Amyotrophic lateral Sclerosis.

References

[1] Johns MW (1991) A new method for measuring daytime sleepiness: The Epworth Sleepiness Scale. Sleep 14:540–545.

[2] Cedarbaum JM, Stambler N, Malta E et al. (1999) The ALSFRS-R: A revised ALS functional rating scale that incorporates assessments of respiratory function. J Neurol Sci 169:13–21.

doi: 10.1016/S0022-510X(99)00210-5.
